# The mitochondrial genome of a social aphid, *Pseudoregma bambucicola* (Hemiptera: Aphididae: Hormaphidinae)

**DOI:** 10.1080/23802359.2019.1622470

**Published:** 2019-07-10

**Authors:** Hui Zhang, Jun Deng, Qian Liu, Xiaolei Huang

**Affiliations:** State Key Laboratory of Ecological Pest Control for Fujian and Taiwan Crops, College of Plant Protection, Fujian Agriculture and Forestry University, Fuzhou, China

**Keywords:** Mitogenome, gene order, gene rearrangement, phylogeny

## Abstract

In this study, the complete mitochondrial genome of the social aphid *Pseudoregma bambucicola* was assembled and annotated. The genome spans 16,631 bp in length with a high A + T content (85.1%), containing 13 protein-coding genes, 22 tRNA genes, two rRNA genes, one control region locating between *srRNA* and *tRNA^Ile^*, and a repeat region located between *tRNA^Glu^* and *tRNA^Phe^*. All the tRNAs except *tRNA^Cys^* and *tRNA^Ser(gct)^* exhibit the typical clover-leaf like structures. The lengths of *lrRNA* and *srRNA* are 1301 bp and 758 bp, respectively. The order of genes between *COIII* and *tRNA^Glu^* exhibits significant difference for *P. bambucicola*. A phylogenetic analysis for *P. bambucicola* and 22 other aphids based on 13 protein-coding genes is also presented.

The aphid *Pseudoregma bambucicola* (Hormaphidinae: Cerataphidini) is an important pest of *Bambusa* bamboos in subtropical areas. This species can produce polymorphic nymphs with behaviour differentiation and some first instar nymphs behave as soldiers to protect their clones (Sakata and Itô 1991). *Pseudoregma bambucicola* belongs to the tribe Cerataphidini, in which several aphid genera can produce soldiers. To date, in Hormaphidinae, only one complete mitochondrial genome of *Hormaphis betulae* from the tribe Hormaphidini has been reported (Li et al. 2017). Here, we report the complete mitochondrial genome of *P. bambucicola*, representing the first mitogenome of Cerataphidini.

Samples used for sequencing were collected in June 2017 in Xiamen of Fujian province, China. Voucher specimen (HL20170622-4) was deposited in the Insect Systematics and Diversity Lab at Fujian Agriculture and Forestry University, Fuzhou, China. The mitogenome was sequenced on an Illumina platform and assembled using NovoPlasty v. 2.7.1 (Dierckxsens et al. 2017). Then, we manually annotated the genome with the MITOS Webserver (Bernt et al. 2013). The sequence has been deposited in GenBank (accession number: MK847518).

The total length of *P. bambucicola* mitogenome is 16,631 bp, longer than majority of published aphid mitogenomes. The nucleotide composition is typically A + T biased (85.1%) with similar patterns also found in other aphids (Wang et al. 2015; Ren et al. 2016; Ren and Wen 2017; Chen et al. 2019; Hong et al. 2019; Voronova et al. 2019). The mitogenome contains 13 protein-coding genes (PCGs), 22 tRNA genes (tRNAs), two rRNA genes (rRNAs), and one control region. Fifteen tRNAs and nine PCGs are located on the forward strand (J-strand) while the remaining genes are transcribed on the reverse strand (N-strand). The mean length of tRNAs is 65 bp, ranging from 52 bp to 73 bp. All 22 tRNAs exhibit the typical clover-leaf like secondary structures except *tRNA^Cys^* and *tRNA^Ser(gct)^*. For the two rRNAs, *lrRNA* (1301 bp) is located between *tRNA^Leu(tag)^* and *tRNA^Val^*, and *srRNA* (758 bp) between *tRNA^Val^* and the control region. The control region (709 bp) with a high A + T content (95.1%) is located between *srRNA* and *tRNA^Ile^*. In addition, a long repeat region between *tRNA^Glu^* and *tRNA^Phe^* also exists, which was thought unique to some aphid lineages (Wang et al. 2013, 2015). However, this region is absent in the *H. betulae* mitogenome (Li et al. 2017). Previous studies revealed that the gene order of most aphid mitogenomes, including *H. betulae*, are identical to that of inferred ancestral arrangement of insects (Wang et al. 2013). However, six genes (J-strand) between *COIII* and *tRNA^Glu^* have undergone significant rearrangements in *P. bambucicola*.

A phylogenetic analysis based on 13 protein-coding genes from *P. bambucicola* and 22 other aphids with complete mitogenomes was undertaken using *Adelges laricis* as outgroup (Figure 1). A maximum-likelihood phylogenetic tree was reconstructed using IQ-TREE (Nguyen et al. 2015), which showed the two Hormaphidinae species, *P. bambucicola* and *H. betulae*, clustered together with strong support. However, some basal nodes with low supports masked the clear relationships between some subfamilies.

**Figure 1.**
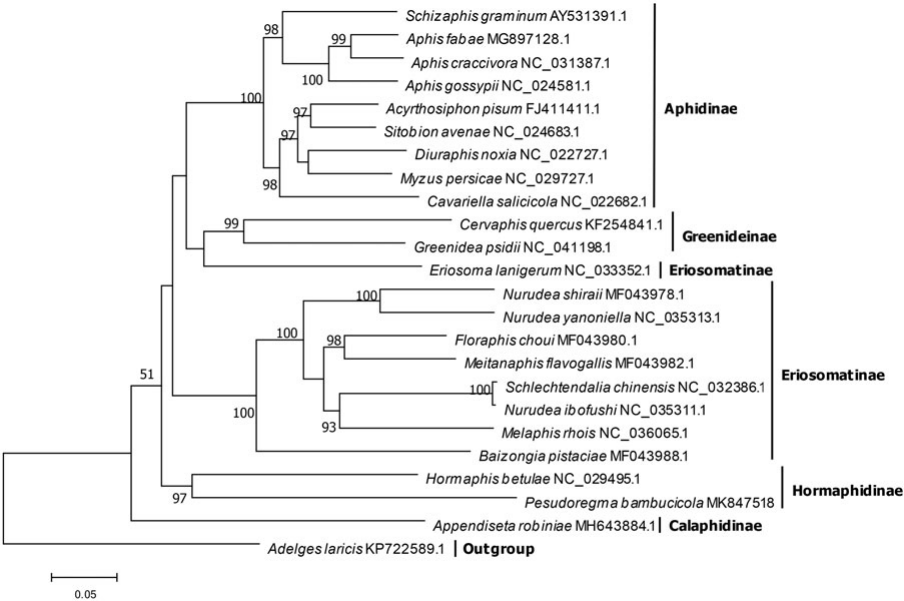
The maximum likelihood tree of *P. bambucicola* and 22 other aphids based on 13 PCGs. Numbers above the branches indicate the bootstrap support values, and values lower than 50 are not shown.

## References

[CIT0001] Bernt M, Donath A, Jühling F, Externbrink F, Florentz C, Fritzsch G, Pütz J, Middendorf M, Stadler PF 2013 MITOS: improved de novo metazoan mitochondrial genome annotation. Mol Phylogenet Evol. 69:313–319. 22982435 10.1016/j.ympev.2012.08.023

[CIT0002] Chen J, Wang Y, Qin M, Jiang LY, Qiao GX 2019 The mitochondrial genome of *Greenidea psidii* van der Goot (Hemiptera: Aphididae: Greenideinae) and comparisons with other *Aphididae aphids*. Int J Biol Macromol. 122:824–832. 30389524 10.1016/j.ijbiomac.2018.10.209

[CIT0003] Dierckxsens N, Mardulyn P, Smits G 2017 NOVOPlasty: de novo assembly of organelle genomes from whole genome data. Nucleic Acids Res. 45:e18 28204566 10.1093/nar/gkw955 PMC5389512

[CIT0004] Hong B, Zhang F, Hu ZQ, Zhao HY 2019 The complete mitochondrial genome of *Indomegoura indica* (Hemiptera: Aphididae). Mitochond DNA B Res. 4:882–883.

[CIT0005] Li YQ, Chen J, Qiao GX 2017 Complete mitochondrial genome of the aphid *Hormaphis betulae* (Mordvilko) (Hemiptera: Aphididae: Hormaphidinae). Mitochondrial DNA A DNA Mapp Seq Anal. 28:265–266. 26713493 10.3109/19401736.2015.1118071

[CIT0006] Nguyen LT, Schmidt HA, Von Haeseler A, Minh BQ 2015 IQ-TREE: a fast and effective stochastic algorithm for estimating maximum-likelihood phylogenies. Mol Biol Evol. 32:268–274. 25371430 10.1093/molbev/msu300 PMC4271533

[CIT0007] Ren ZM, Bai X, Harris AJ, Wen J 2016 Complete mitochondrial genome of the *Rhus* gall aphid *Schlechtendalia chinensis* (Hemiptera: Aphididae: Eriosomatinae). Mitochond DNA B Res. 1:849–850. 10.1080/23802359.2016.1241678 PMC7799637 33473653

[CIT0008] Ren ZM, Wen J 2017 Complete mitochondrial genome of the North American *Rhus* gall aphid *Melaphis rhois* (Hemiptera: Aphididae: Eriosomatinae). Mitochond DNA B Res. 2:169–170. 10.1080/23802359.2017.1303345 PMC7800657 33473755

[CIT0009] Sakata K, Itô Y 1991 Life history characteristics and behaviour of the bamboo aphid, *Pseudoregma bambucicola* (Hemiptera: Pemphigidae), having sterile soldiers. Insect Soc. 38:317–326.

[CIT0010] Voronova NV, Warner D, Shulinski R, Levykina S, Bandarenka Y, Zhorov D 2019 The largest aphid mitochondrial genome found in invasive species *Therioaphis tenera* (Aizenberg, 1956). Mitochond DNA B Res. 4:730–731.

[CIT0011] Wang Y, Chen J, Jiang LY, Qiao GX 2015 Hemipteran mitochondrial genomes: features, structures and implications for phylogeny. Int J Mol Sci. 16:12382–12404. 26039239 10.3390/ijms160612382 PMC4490450

[CIT0012] Wang Y, Huang XL, Qiao GX 2013 Comparative analysis of mitochondrial genomes of five aphid species (Hemiptera: Aphididae) and phylogenetic implications. PLoS ONE. 8:e77511 24147014 10.1371/journal.pone.0077511 PMC3798312

